# The Dental Educational Environment of Online and Blended Learning during COVID-19, and the Impact on the Future of Dental Education

**DOI:** 10.3390/dj11020041

**Published:** 2023-02-07

**Authors:** Mai E. Khalaf, Hassan Ziada, Neamat Hassan Abubakr

**Affiliations:** 1 Department of General Dental Practice, Faculty of Dentistry, Kuwait University, Safat 13110, Kuwait; 2 Department of Clinical Dental Sciences, School of Dental Medicine, University of Nevada, Las Vegas, NV 89106, USA; 3 Department of Biomedical Sciences, School of Dental Medicine, University of Nevada, Las Vegas, NV 89106, USA

**Keywords:** dental, education, blended learning, online learning, self-perception

## Abstract

Blending face-to-face and online learning should create a focused environment that supports deep and meaningful teaching and learning that engages learners in a more active and collaborative educational experience. The present study aimed to evaluate students’ online and blended learning educational environment self-perception at the Faculty of Dentistry, Kuwait University, during the COVID-19 pandemic. Methods: Undergraduate dental students who participated in blended learning with online lectures were invited to participate. The sample was a non-probability convenient sample, which included all clinical dental students invited to participate, who were enrolled in the fifth, sixth, and seventh (clinical year) years. All 69 students in these three clinical years were invited to participate. Electronic consent to participate and a self-administered questionnaire of two parts were completed. Part one of the questionnaire utilized the five subscales of the Dundee Ready Educational Environment Measure (DREEM) questionnaire; part two was developed in addition to evaluate the online teaching and learning subscales. Results: Descriptive statistics and analyses of variance were performed; Pearson correlations were made between the additional supplemental online teaching subscale and the original DREEM subscales. The mean students’ perception of the teacher was high, followed by the academic self-perception and then the learning perception. Students’ social self-perceptions had the lowest reported scores. Students’ perceptions varied by year of education in all subscales except for the online domain. In comparing all domains (DREEM and the online component), graduating students (final year) had a more favorable perception than other students. Conclusions: Within the limitations of the present study, online and blended learning were positively perceived, excluding the social self-perception and the perception that the online teaching time was not well used.

## 1. Introduction

Dental education integrates didactic, laboratory, and clinical psychomotor skill development to eventually lead to the clinical competence of a graduate who can practice independently. This student-centered multi-faceted pedagogical and clinical integration is challenging for students and educators. In this educational journey, the students learn the underpinning theories and principles of the related sciences and develop psychomotor skills while maintaining the desired level of professionalism. Other elements of this educational journey are the growth and development of problem-solving abilities, critical thinking, reflective practice, and the ability to build dynamic relationships with patients and teams [1]. This should instill a desire to improve and update skills in a life-long learning process. Although many technologies became prominent during the COVID-19 pandemic, such technologies were suggested prior to the COVID-19 era. The introduction of these technologies to dental education should also improve patient health care. This will likely lead to a more person-centered approach with the desired self-reflection, learning, and patient care [2].

The coronavirus (COVID-19) pandemic in early 2019 globally impacted all elements of life and placed challenges on academics in delivering their mission and on students to attain and maintain the required knowledge and skills [3]. In academia, learning technologies and emergency remote teaching were used, and the indications support these learning technologies and their possible continuum in dental education [4]. Nevertheless, we have to take into consideration that virtual learning may require self-discipline and responsibility more than the face-to-face mode would [4].

At Kuwait University, Faculty of Dentistry, as in other schools, the management crisis during the COVID-19 pandemic involved the integration of various elements of information delivery and clinical skills development by limiting didactic education to online instruction via multiple online platforms and a planned gradual resumption of onsite laboratory and clinical sessions, a combination that can be regarded as “blended learning”. 

Blended learning has demonstrated better knowledge acquisition than traditional learning in health education [5]. It also seems to have a consistently positive effect and has been reported as more effective than, if not as effective as traditional teaching for knowledge acquisition in the health professions [6,7]. As vaccination rates increase and countries move forward in the resumption of face-to-face activities, institutions are in a position to evaluate the integration of alternative educational methodologies, assess their impact, and evaluate the prospects of integrating them into the future educational process. Other factors that encourage the return to normality include the desire to reduce or eliminate social distancing and overcome the general perception of missed or lost learning. Furthermore, the economic pressures to return and the need for independence by individuals, as well as their need to eliminate the reliance on governments or authorities dictating or limiting movements and behaviors, participate in this.

Should educational institutions put the experience behind them or learn from that experience and consider adopting modification measures used during the pandemic in future dental education, one of which is blended learning? To answer this question, we should evaluate the students’ voices on this experience. The educational environment impacts students’ learning, satisfaction, and success, and it is important to continue to address issues in their feedback and evaluate how they perceive their educational environment. Students tend to process their learning experiences differently; therefore, investigating how they perceived online and blended learning educational environments during the pandemic is essential to evaluate the quality of teaching and assess areas where improvements can be made to advance and increase the efficiency of students’ learning [8].

We used the Dundee Ready Education Environment Measure (DREEM) and a developed supplementary questionnaire to address the online domain. It has been effectively used to evaluate the differences between how students perceive their educational environment and what they like in this experience [8]. In recent studies using DREEM during the COVID-19 pandemic, Ref. [9] found that their virtual interactive platforms for teaching and learning were perceived to be effective by predoctoral medical students [9]. Ref. [10] evaluated the DREEM subscale scores before and after the onset of COVID-19 among premedical students and found that they perceived their education environment more positively after the experience that followed the onset of COVID-19; however, the social self-perception domain improved the least [10].

The hypothesis is that the students would favor online and blended learning as a platform for dental education. The present study aimed to evaluate student self-perception of online and blended learning educational environments in aspects related to their teachers, social self-perception, and academic self-perceptions, using the DREEM and a supplementary questionnaire. 

## 2. Materials and Methods

A descriptive cross-sectional design was used to evaluate clinical predoctoral/undergraduate dental students’ online and blended learning educational environment at the Faculty of Dentistry, Kuwait University. The Faculty of Dentistry in Kuwait implements a seven-year dental curriculum. First, four years are biomedical courses taken jointly with the medical students at the Faculty of Medicine. This is followed by three years of clinical education and training. The number of students accepted per year is approximately 28 students.

The Dundee Ready Education Environment Measure (DREEM) used in this study is a validated measure that evaluates the educational environment, designed specifically for medical schools and other healthcare professions. It was developed with an international perspective as a generic instrument that is not regionally or culturally specific [11]. It is a multicultural and independent valuable instrument for evaluating the educational environment and student learning, as well as providing reliable feedback on the strengths and weaknesses of the educational environment [12].

### 2.1. Ethical Considerations

Ethical approval was obtained following a study protocol submission to the Joint Committee for the Protection of Human Subjects in Research of the Health Science Center, Kuwait. Confidentiality and voluntary statements were included with a cover letter explaining the study’s purpose. The participants were also guaranteed anonymity and confidentiality and that no participant information would be identified or collected.

### 2.2. Recruitment and Sample

The sample was a non-probability convenient sample, which included all clinical dental students at Kuwait University Faculty of Dentistry, the only undergraduate dental teaching institution in the country. Those who participated in online and blended learning during the pandemic were invited to participate, which included students enrolled in the fifth, sixth, and seventh (clinical year) years. All 69 students in these three clinical years were invited, and only those who provided voluntary and informed consent were included in this study. 

### 2.3. Procedure 

Students who agreed to participate were invited to complete an electronic self-administered questionnaire in the English language consisting of two parts. They were requested to complete all questions in both questionnaires. This ensured a 100% item response rate in the analyzed data. A missing response would result in excluding the participants and their responses from the study and the analysis.

Part one of the questionnaire employed the five original subscales of the DREEM questionnaire (Dundee Ready Educational Environment Measure) developed by Roff et al. [13]. This comprises 50 questions that evaluate students’ perceptions in a five-domain format. These included the perception of learning in 12 questions, the perception of their teachers in 11 questions, academic self-perception in 8 questions, the perception of the atmosphere in 12 questions, and the social self-perception in 7 questions. For negatively worded items, reverse coding was also used. Adding up all items, a total DREEM response score of 200 would be achieved. Each item of the questionnaire used a 5-point Likert scale ranging from 0, which is strongly disagree, 1 = disagree, 2 = uncertain, 3 = agree, and 4 = strongly agree. The first three score domains would calculate a maximum of 48, 32, and 48. The total domain scores were calculated and converted into percentage scores to permit interdomain comparisons. Higher scores suggest learners’ more positive determination of the educational environment [14].

If negative responses in items #4, 8, 9, 17, 25, 35, 39, 48, and 50 were present, the direction of the scale would be reversed. DREEM also allows for individual items’ mean scores to be further evaluated to assess detailed strengths and weaknesses within the educational environment. Part 2 was a supplementary questionnaire specifically developed to relate to the online domain of the blended learning environment. This was designed similarly to the DREEM with a 5-point Likert response scale to measure specific issues associated with the virtual/online component.

The supplementary questionnaire investigated the advantages and disadvantages related to online delivery. These included the virtual online learning environment, if it allowed more efficient use of students’ study time, if they could acquire more in-depth knowledge, if it was easier to take notes and the absence of disturbance by other students, and the better concentration on the material being presented. It also evaluated the disadvantages of the potential of only superficial knowledge being gained in an online environment and the reduced interaction with fellow students and teachers. Additionally, if they felt they had limited class time, if they experienced a sense of isolation, the inability to ask questions, the dependency of the environment on the internet connection and the requirement of access to a mobile smart device. Initially, 20 students completed this questionnaire to evaluate internal consistency reliability. This was made using Cronbach Alpha, one of the standard methods employed for this purpose. This supplementary domain reported internal consistency reliability, with a Cronbach Alpha value of 0.723.

### 2.4. Statistical Analysis

Descriptive statistics were used, and the underlying assumption of normality was confirmed using z-scores before conducting comparative analyses; by dividing skewness by the standard error of skewness, a z-score within +/−3.29 is indicative of a normal distribution [15]. Moreover, normality and the absence of outliers were confirmed by visual inspection of histograms. Internal consistency reliability was confirmed using Cronbach’s alpha coefficients. 

Furthermore, parametric tests were used to analyze ordinal data since they are usually more robust than nonparametric tests; for example, in Likert responses, even when statistical assumptions (such as normality of data distributions) are violated [16]. Pearson correlations were also conducted between the supplemental online subscale and the original DREEM subscales. One-way analyses of variance and post hoc pairwise comparisons with Bonferroni adjustment were conducted between each academic year. The level of statistical significance was set at 0.05.

## 3. Results

A total of 60 out of the 69 total number of students (a response rate of 87%) completed all two-part online questionnaires. Their demographic characteristics and details regarding their use of online learning are summarized in Table 1.

**Figure 1:**
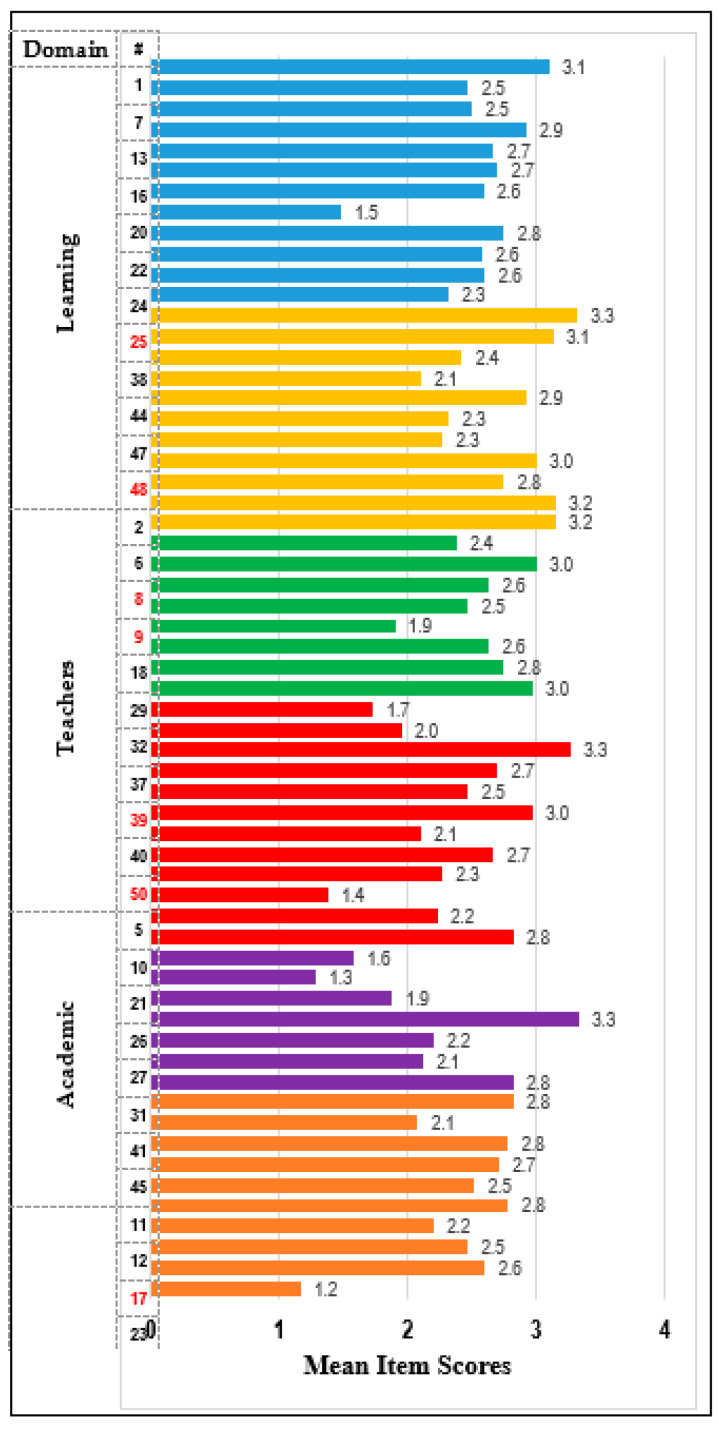
Mean scores for DREEM and the online domains (reverse-scored items are noted in red).

All students responded to the 50 DREEM questions on the educational environment and the additional supplementary questionnaire regarding the online component. Six subscales and an overall total score were computed for the DREEM. The supplemental questionnaire assessed the students’ perceptions concerning the online component. Responses regarding drawbacks were reverse-scored and added to the five questions regarding the advantages for an overall score. Item means are presented in Table 2 and displayed in Figure 1. 

All scales were normally distributed (z < ±1.2) and internally consistent (α ranged from 0.73 to 0.94). The mean DREEM score was 125.71, and adding the supplementary online component made an overall mean score of 149.08. The mean students’ perception of learning was 63.89%, that of the teacher was 69.40%, the academics’ was 64.78%, the atmosphere’s was 59.47%, social self-perception’s was 54.35%, and the online component’s was 60.32%.

All five original DREEM subscales were highly correlated (Table 3). Pearson inter-correlations between the supplemental online subscale and the original DREEM subscales showed small but significant relationships between the online subscale and three of the DREEM subscales (Learning, Teachers, and Academic). The strongest relationship was with the third DREEM subscale, which is the student’s academic self-perception (r = 0.38, *p* = 0.003).

**Figure 2:**
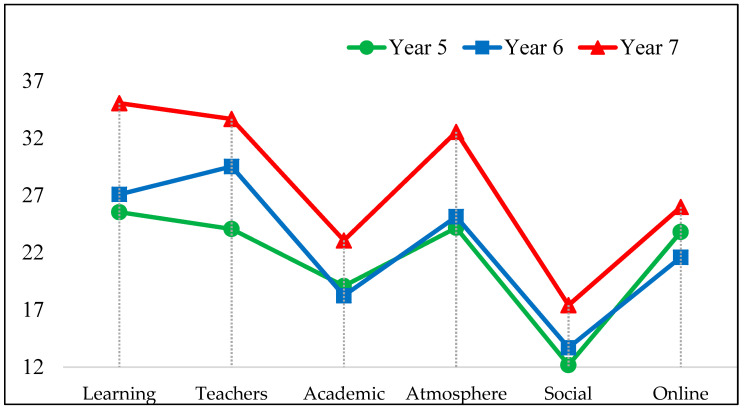
Mean subscale scores by academic year.

The scales were further compared across the three academic years using a one-way analysis of variance. The results in Table 4 and Figure 2 show students’ perceptions to have varied significantly by year in all subscales except for the online component. Post hoc pairwise comparisons with Bonferroni adjustment between each academic year revealed that students’ perceptions in years 5 and 6 did not differ significantly, except for the perceptions regarding the teachers. However, the students’ perceptions in year 7 (Final) in the five original DREEM domains were significantly more favorable than those from the two former years.

## 4. Discussion

This study evaluated student-reported elements of online and blended educational environment in aspects related to teachers, academic self-perception, and the social self-perception. This should help in evaluating the consideration of the integration of this teaching mode in the long-term planning of optimizing the teaching of the dental curriculum. 

Overall, dental students in their clinical years of education reported an overall positive perception of online and blended learning; issues reported were concerns about the quality of the use of teaching time. 

### 4.1. Students’ Perception of Learning

There are continuous global educational attempts to improve the quality of teaching and the learning environment, and students’ voices have been used as reliable sources for this information; accordingly, more focus has been dedicated to pursuing students’ opinions [17]. These evaluations can give us insight into the efficiency and perceived benefits from the perspective of dental students. However, correlating their perceptions with their outcome assessments would have also been beneficial. It also permits dental educators to evaluate where they stand on current traditional face-to-face teaching and help them integrate new teaching and learning modalities into dental education, particularly if it is perceived as more effective or more acceptable by students. The DREEM instrument has been used and proved appropriate for evaluating the educational environment in medical and dental education [18,19].

The educational environment includes the organization, the teachers’ behavior, and the adopted teaching philosophy and methodology. It significantly influences the program’s efficacy, curriculum, and educational outcomes [20]. In this study, the student’s perception of online and blended learning was similar to the outcomes reported by Chew and Sim [14] among fourth-year medical students undergoing psychiatry rotation, which was positive, except also similar to this study, for the utilization of teaching time to good use and the students’ social self-perceptions. This included self-reported satisfaction with class participation, finding the teaching current, research-based, student-centered, and focused on elements that help develop confidence and competence. A systematic review sees blended learning potentially improving clinical training and knowledge acquisition among medical students [3]. Studies conducted in several countries found that dental students’ perceptions were generally favorable and that it was an adequate model and user-friendly [21]. Studies from India, Nepal, and Sri Lanka reported that dental students positively perceived an online teaching and learning environment, and that an internet-based component in teaching provides ease of accessibility and a stress-free environment, which could have led to this overall positive perception [22,23,24].

In this study, students’ perceptions of their educational environment in year five scored the lowest average, particularly on the “well use of teaching time”. This may indicate that newer students entering the clinical part of the education would be more critical of the virtual component of the blended learning model of teaching delivery, with the mean scores increasing as students progressed into their clinical education. In agreement with the present study, Refs. [20,25] found that among medical students, the perception of the educational environment varied between individual years of study [20,25].

The current study also found that when comparing the five original DREEM domains and the online domain, the students in the graduating class (the final year) had statistically significantly more favorable perceptions than those in earlier years. This can indicate that, as students progress in their education and increase their clinical experience, they seem to gain more confidence and better perceive their educational environment and surroundings. This agrees with [5], according to whom blended learning may positively affect knowledge acquisition and should be viewed as a promising approach in healthcare education [5]. 

The educational environment significantly impacts students’ learning, satisfaction, and well-being. It prepares them for independent practice and student satisfaction [26,27]. Students’ social self-perceptions were the area that seemed to have the lowest reported scores amongst students across the three clinical years of the study, with the younger cohort again reporting the lowest scores. Ref. [10] found that the social self-perception domain improved the least [10]. Students’ social self-perception has been linked to stress, consistently recognized as a significant contributing aspect to poor student performance [19]. However, in Australia and Europe, stress in dental schools has been attributed to exam anxiety; in Canada and Europe, to finance, although the most attributed was to stress [28,29,30]. However, stress may have been a factor that has been prominent during the period of the COVID-19 outbreak and may have impacted students’ perceptions in this study. Nevertheless, a study from India reported stress to be mainly a teacher-related issue [21].

### 4.2. Limitations 

The study had limitations; the findings are based on students’ self-reported perceptions, which can be associated with self-reporting bias based on some students’ feelings when the survey was conducted. Furthermore, students’ perceptions may have been impacted by external variables, for example, the circumstances in their homes or the country regarding the pandemic. That may have also impacted the overall student feelings and attitudes, influencing the responses to the questions in the survey. Moreover, the sudden change to online and blended learning from traditional face-to-face learning may have impacted students’ perceptions since neither they nor their teachers were prepared for it [31]. Additionally, and to be considered, DREEM’s overall and subscale scores, while they gauge the educational environment, they may, however, mask the presence of other educational problems [30]. Moreover, the study evaluated psychometric properties as validity; but these may not relate to the instrument itself but rather part of an account of the results produced from the related study. Consequently, if and when the same instrument is applied to a different population, their experimental questions and results need to be analyzed psychometrically for that particular population. In this regard, we understand that Cronbach’s alpha is only meant to test the internal consistency reliability of the measure and that there are several other aspects of reliability and validity which can be assessed. However, these assessments are beyond the scope of the current study, and we are satisfied that the DREEM is valid and reliable in addressing the DREEM’s psychometric properties [32]. Furthermore, responses regarding drawbacks were reverse-scored and added to the five questions; Ref. [33] reported that such reversed and negatively phrased items present scalability problems [33]. However, this lack of strong support for the scalability was related to a sample of undergraduate physiotherapy students and not dentistry, and perhaps the hands-on component of dentistry may not have the same problem.

### 4.3. Implications and Future Research

The immediate impact of the pandemic has been reported, but the medium-to-long term impact is unclear [3]. This study reports the outcomes in a “forced by the pandemic” environment of online learning blended with practical and clinical activities through social distancing; however, it showed a positive educational environment that seems to have been perceived positively by students. Therefore, online and blended learning may be considered a formal and validated educational methodology in dentistry. One of its advantages is that the range of knowledge that our students need to acquire, and the skills they need to master are increasing while the time frame for their years of education remains the same; hence, blended learning seems a reasonable approach to consider to overcome these and other challenges in dental education [7]. 

Online and blended learning adoption poses another advantage: transferring knowledge to many recipients and accommodating different learning styles [16]. However, blended learning may need more effort to implement than the traditional model; it requires the right attitude, significant financing, and highly motivated teachers and students for its successful implementation. Qualitative studies can explore the benefits gained from online and blended learning and if it is favored by students to move to an online and blended mode of learning in the future. The same authors of this study are currently analyzing such qualitative data. Furthermore, participants had limited experience with online learning at the start of the pandemic; however, a good experience has been developed to discuss and reflect on online and blended learning experiences in a qualitative study. Moreover, multicenter studies can also evaluate the move toward online and blended learning that considers students’ and teachers’ perceptions. Furthermore, teledentistry was reported to be a possibility to help dental patients and dentists, during the time of the COVID-19 pandemic; future studies should also evaluate its validity post COVID-19 pandemic [34]. Finally, continuing research should help us move forward to provide innovation in dental education. We should also continue investigating how to increase the involvement with our students and colleagues, since it will have a positive impact on the educational environment [3].

## 5. Conclusions

Dental students in their clinical years of education (final three years) at Kuwait University Faculty of Dentistry reported an overall positive perception of blended learning, except for social self-perception and teaching time which was not well used during blended learning. The transformation of dental education from face-to-face to blended learning formats is a viable option for future dental education.

## Figures and Tables

**Table 1:** Sample characteristics.

		Frequency	Percent
Years of age			
	18–20	1	1.7
	21–23	24	40.0
	24–26	34	56.7
	27+	1	1.7
Academic year			
	5th	11	18.3
	6th	20	33.3
	7th	29	48.3
Gender			
	Female	57	95.0
	Male	3	5.0
Device used in online lectures			
	Laptop	33	55.0
	Tablet	25	41.7
	Smartphone	2	3.3
Location of attendance			
	Home	58	96.7
	Another place	2	3.3

**Table 2:** Summary statistics for the study scales.

Scales	*Mean*	*SD*	*Skewness*	*SE*	*z*	*α* *
I Students’ perception of learning (48)	30.67	7.38	−0.10	0.31	−0.33	0.84
II Students’ perceptions of teachers (44)	30.54	6.57	−0.33	0.31	−1.08	0.85
III Students’ academic self-perceptions (32)	20.73	4.88	−0.07	0.31	−0.22	0.76
IV Students’ perception of atmosphere (48)	28.55	8.64	−0.15	0.31	−0.48	0.86
V Students’ social self-perceptions (28)	15.22	4.89	−0.20	0.31	−0.66	0.73
VI Students’ perceptions of online component (40)	24.13	8.96	−0.34	0.31	−1.10	0.88
Total score (240)	149.08	32.53	−0.03	0.31	−0.09	0.94

* Cronbach’s alpha.

**Table 3:** Pearson intercorrelations for the supplemental online subscale and the original DREEM subscales.

	II		III		IV		V		V1	
Learning	0.75	***	0.80	***	0.75	***	0.73	***	0.26	*
Teachers			0.60	***	0.64	***	0.54	***	0.19	
Academic					0.83	***	0.81	***	0.38	**
Atmosphere							0.77	***	0.24	
Social									0.29	*
Online										

* *p* < 0.05, ** *p* < 0.01, *** *p* < 0.001.

**Table 4:** Analysis of variance on the subscales across the three clinical years.

	Year 5 (n = 11)		Year 6 (n = 20)		Year 7 (n = 29)			
	Mean	SD	Mean	SD	Mean	SD	F	P
Learning	25.5	6.2	27.1	6.3	35.1	5.9	19.59	<0.001
Teachers	24.1	6.5	29.5	4.3	33.7	6.0	23.66	<0.001
Academic	19.1	3.9	18.3	3.9	23.1	4.8	6.56	0.013
Atmosphere	24.2	7.1	25.2	7.6	32.6	8.2	9.11	0.004
Social	12.2	5.8	13.7	3.9	17.4	4.2	11.08	0.002
Online	23.8	9.8	21.6	9.7	26.0	7.9	0.48	0.493
Total score	126.2	26.3	135.2	25.7	167.3	29.1	17.84	<0.001

## Data Availability

The datasets used and/or analyzed during the current study are available from the corresponding author upon reasonable request.
